# Pure Milk

**Published:** 1867-06

**Authors:** 


### PURE MILK.

Some years ago, a few gentlemen of New York city, impressed with the conviction of the pernicious effects on a family of growing children, induced by the use of milk from cows fed with the "swill" of distilleries, especially when connected with the barbarous practice of confining the animals in small stalls, reeking with filth of every description, and milking them until the day of their death by literal rottenness of different portions of their bodies, made an effort to have milk sent to them every day from the farm-houses of personal friends in the same neighborhood. In a very short time, the inquiry was made almost every day by friends and visitors who chanced to call at meal time, "where do you get such delicious milk? we have never tasted such in New York before." The story was repeated and then came the solicitation, "Can't you admit me into the club?" In this way, the few soon became an association of many and a company was formed; but for the purpose of securing the continuance of a supply of milk, rich, fresh, and pure from farm-house cows, it was necessary to guard against the wrong doing of those who delivered the milk to the different dwellings, and it was done in this way: each family was supplied with two tin cans with brass labels, and a cover fastened with a lock, and two keys, one for the use of the superintendent, who saw that each can was filled and locked in his presence, the other was kept by those who received the milk; hence, the great boon of pure farm-house milk in New York City. We have been using it for some ten years, meanwhile the club has grown so large under the judicious and watchful management of S. W. Canfield, Esq., that two offices are required to transact the business; one for down-town, at No. 148 Tenth st., just east of Broadway, and the other for the up-town customers, in Broadway, corner of 37th street, proving the old adage, that "honesty is the best policy," and, that whoever will engage in any legitimate business in New York, and follow it up, fair dealing being the ruling principle, will eventually succeed, not only in getting the peoples' money, but getting their confidence and respect, which is worth a great deal more.

### TEA AND COFFEE.

Of the large number who have dealt in swill milk, and in selling milk and water for the unadulterated article, and have failed at last, dying in poverty, or have gone to the penitentiary in disgrace, as the legitimate result of their pernicious practices, willing to risk the destruction of the constitutions and lives of unoffending infants and children, in supplying them with the milk of diseased and dying cows, no more need be said; they are reaping their reward; but it is very natural to pass from milk and water to tea and coffee, as milk is almost universally used in them. Tea admits of several injurious adulterations, and while families have been poisoned in Brooklyn by the use of coffee purchased already ground, several "great" companies have sprung up in New York City of late, all of them, it is said, being composed of one man, like a certain "Howard Association," for the benefit of frightened, green young men. It is the business of an editor to make experiments, and record his observations for the benefit of those who pay him for instruction, so we called at one of the "great" companies one day, not long ago, and bought some ground coffee, the very best they had in the store, warranted as pure as anything else in the establishment; a paper was handed with the purchase, setting forth the anxiety of this "great company" of one man, for the health and thrift of the people; the great efforts they had been making, and the small amount of profit they were willing to work for, and how they could sell pure ground Java coffee for less than the green article could be purchased, it being known that from six to sixteen per cent. was lost in burning or roasting, besides the expense of roasting, grinding, paper, twine, shop rent, &c., &c. How they could do all this was made as plain as a pike staff, thus:—"You see, sir, that between John Chinaman who raises the tea, or the Java planter and you who drink the article, there are six or eight middle men who make a living; now, to save all this, we purchase our articles from the planter, who, himself, delivers it on board of our vessels, from which we bring it in our own drays to this store; our wives, sons and daughters roast, grind and tie up, so that we are at no outlay at all, and this is just exactly the way the milk got into the cocoa-nut and the man got out of the bung hole, after he had finished the barrel on the inside. Well, we went home wondering. The coffee was boiled, the "grounds" taken out and spread in the sun to dry, and a microscope was brought into requisition, with the following results; the real coffee was black and hard almost as sand, but there were particles larger, lighter, and so soft, as to mash up like a jelly under the knife; this was rye or other grain. We advise our farmer readers not to pay forty cents a pound for roasted, ground rye; they can probably raise it for a cent a pound. "They say" that this great company of one, owning several great offices in different parts of New York, has already made a large fortune. We would like some moral philosopher of great skill in deciding nice points to say, on the presumption that the sentiment uttered by the immortal Watts is still true;

> Let little children be so wise,
> To speak the truth and tell no lies;
> For liars' portion it is to dwell
> Forever down below.

If a man tells one deliberate lie is he a liar?

If a man has to go below for telling one lie, how long must that plummet be which will reach him who repeats that lie millions of times every day to millions of people? But pure milk reminds us of

FRESH EGGS, and our adventures in search of them this spring. A box was sent us by express; on delivery at our door we discovered that one end of the box was yellow, the other white; this suggested that the articles inside were not in a good state of preservation. We suggested to the man the propriety of opening the box first, to see if all was right before paying a heavy freight on a costly box of broken eggs. "It's agin the company's rules to have a box opened until it is paid for."

"But suppose it is damaged." "Why, then you goes to the company and makes reclamation." "Which being interpreted, means, when all charges are paid then I may whistle for the eggs." "Just so sir, 'xactly." "Well, you can take the box back." So in a day or two we thought we would take a ride of six miles there and back, and make some discoveries about the morals of express companies, and the value of broken eggs. We asked for the head of the proper department who was all smiles and accomodations. "Just pay charges, open the box, see the damage done, and make ‘reclamation,’ and it will be all right in the morning.” Next day it was, Monsieur Tomson come again in the shape of the box; the yellow coat had all been removed, indicating that the eggs had been mended, and that Richard was himself again, except that about twenty per cent. extra was added for freightage the second time; which was paid without a word. Next day the box was opened, and over one fourth of the eggs were broken; others being present as witnessess. Being somewhat interested in finding out whereunto these things would grow, we made a second voyage of discovery all down Broadway below Trinity church. But the man was not to be found who talked about “reclamation.” Mr. Fare was introduced to us. To save time and words, a statement was handed in, in two lines, so many whole eggs, so many broken ones, so many per invoice. The man at the desk then put on a serious air, very business like, and began to argue the case. We said nothing but listened to the following verbatim statement. “You see, sir, the presumption is the box was filled with eggs, and that in coming such a distance a few would be necessarily broken, the contents escape and there is a vacuum; this vacuum being made, the remaining eggs had more room to fly around and be broke too, hence it is reasonable to infer that the eggs were broken in consequence of your sending them all the way down from Forty-third street to sixty-five and a half Broadway, hence, as you broke the eggs yourself, (constructively) we ought not to be required to pay for them, at least, only for one or two—How much do you ask?

Now, as we didn't sell eggs, and don't believe in arguing a case, we simply pointed to the written paper; so many sent, so many arrived sound, “you can send me what you think is right.”

That was the last of it, of course. What would the enterprising, hard working, honest Harden say, if he could come out of his grave in New England, and see the profanation of business interests in his name, here, in Gotham.

This is the way a certain class of men do business in New York. We earnestly hope that the New express company in

Broadway, No. 365, cor. of Franklin st., will inaugurate new principles of business conduct, to wit, speed, safety, moderate charges, and a prompt and honorable compensation for injuries done to the property of their patrons. We advise all our readers to give them a trial at once, and continue to patronize them as long as they do right; and let them see that doing right will always command wide patronage in this community. Some express companies make a great ado through the public papers in replacing large amounts lost through them by banks, bankers, and rich corporations, but more than make it up by over charges and other rascality in that vast multitude of cases, where the loosers are poor or obscure, or do not care to lose time for trifling amounts, or wear them out in long and expensive lawsuits. But speaking of pure milk and fresh eggs this May morning, reminds us of

SUMMERING IN THE COUNTRY, which, as done by the masses is an indescribable, an incomprehensible absurdity. Young gentlemen should go afoot in companies, camp out, fish, hunt, swim, sail, row, botanize, or with hammer and satchel study the rocks and read their individual histories from the hours that time began. Mothers and daughters should hie away to some retired farm house, or to some mountain home, and with sun bonnet and plainest calico dresses in shape most convenient for wandering over the hills and far away, or for mounting their ponies and gallop away in gleeful mood for an hour or two every morning, then read awhile and go a berrying or to the dairy or to the kitchen and try their hands at some new receipts in cookery; then after tea go on foot a mile or two away with brothers and beau to a quilting or a singing school or to meet at some neighbor's house for frolic and fun in the abandon of childhood and innocence; these are some of the wise ways of spending a summer in the country as a means of resting the mind, of renovating the brain, and giving new vigor to the whole physical man. But the misfortune is that very few go to the country in summer with such aims and ends. The first and main desire of many is a " good table;" three times a day they want spread before them the richest food, the most enticing viands, the most luxurious delicacies, and if these are not secured, all the advantages of the seaside and the spa fail to avert from the place their unstinted anathemas. If, on the other hand, a retired place in the country is secured, there is nothing to interest them, and the greater part of their waking existence is spent in lounging on a sofa, in lolling in a rocking-chair or in dozy dreaminess on a bed. Once in a while they take a listless stroll through the "grounds" of the establishment, if, indeed, these "grounds" are any thing more than a cow pasture or a weedy "garden" with here and there a morning glory, a hollyhock and a marigold. The morning paper is the greatest event of the day; after that comes "Harper, or Godey, or the Atlantic monthly; but these are too heavy for their light heads, and soon they fall to stretching and yawning, then overpowered by sleep until the gong or steer's horn wakes them up, they feed without relish and without appetite, because they take no exercise, for in the morning the dew is on the grass and they can't walk out, at noon it is too hot, after dinner they are sleepy, and they must have a "nap;" then it is so sultry and dusty that no pleasure can be expected either from a drive or promenade unless it is after tea, and then the night air is the great bugbear. Very soon this inactive, jejune life brings on a loss of appetite, and they expect their host to wake it up by having some new dish; a large part of their time is spent in wondering what will be for the next meal and criticising the last. And thus, after all, spending a summer in the country means an unvarying round of eating lounging, sleeping and listlessness.

This going to the country for the summer is a great need for old and young in the large cities, growing children especially; but the way it is managed generally it is a perfect farce. Just look at the items. You exchange a fine house with large, airy rooms and ample closets for one single apartment, which, by the time you have your trunks arranged, leaves you barely space to turn round; if you want to hang up your coat you must go to the "village" and get a nail. If you desire a drink of water you must go down stairs for it, or more likely, to the kitchen or pump, for in the "parlor" you find that children and nurses have monopolized every "tumbler" and forgot to remove certain remnants of sweet cake from around the edges and the bottom of the glass. If you want a cup of hot water you must go to the cook, and be very polite too, or you won't get it until next day. At nightfall you go groping about with a tallow candle or a kerosene lamp, with its long, tottering chimney, which you are every moment afraid will tilt over, leave you in Egyptian darkness, or blow you, and may be, the whole establishment, sky high, besides spoiling the old woman's carpet. The first morning you come down to breakfast, your mouth fairly waters at visions of sweet butter, fresh eggs and pure milk, but you soon find that the butter might have been sweet a year or two ago, but not now; the very first egg you crack open has a spring chicken in it, but you expected to have spring chickens at a country farm house as a matter of course, and now you have them sooner than you expected; as to the milk, it had been sent for sale to the village the day before, and this remnant was put on the table to prevent its going to waste, and as it was to be mixed with coffee, it made no difference if it did stand around in white spots. The bread! is it the crisp French roll of the city? Nothing of the sort, it is either of a leaden sogginess, or so yellow that it reminds you of an apothecary shop, and you begin to wonder if the entire stock in trade of saleratus of the village druggist was not put into that one plate of biscuits. In a very short time you find that the "variety" of the establishment has run out. You have ham for breakfeast, corned beef for dinner, and sour preserves for supper; then apace come the hot nights; you are in the attic and you fairly sweat in the daytime, and stew and fret and fume in the night, the perspiration streaming at every pore, while the lively musquito keeps up his jubilee; but he soon gets his fill and troubles you never again; at midnight, however, other foes must feed, and you turn and scratch, and scratch and turn until the morning begins to dawn, when exhausted nature falls into the arms of Morpheus as helpless as a baby; but you don't stay fallen thus for a single hour, for the impertinent fly has had nothing to eat all night and is now perfectly ravenous, and in his winding way over face and nose and forehead and lip, searching for a convenient spot to thrust his spear into, he breaks in upon your dreams and rouses you up to the realities of a " summer in the country." Compare such a life with the luxuries of your own city home ; the convenient gas, the iced croton, the hot water always ready at your elbow day or night by the turning of a fawcet, the privilege of roaming from one spacious apartment to another, to the front room in the morning, and rear at night or vice versa, so as to baffle the heating sun ; the spacious hair mattress, springy and cool. At breakfast, the yellow butter, made but yesterday, the luscious cream-cup, the French rolls, and the porter house, with the Times, Herald or Tribune to tell you what was said twelve hours before by Napoleon, or what was done by Bismarck, what Turkey fears, what Russia hopes, and what of progress is making in our own land of promise and of hope. After breakfast you take your walk on dry pavements on the shady side of the street, at midday you keep quiet in the cool of your office or counting-room, and at evening are fanned by the gentle west wind as you take your plate of Fuscell's ice cream at nine o'clock with your friends seated around you, and then retire in the confidence of sweet dreams, and sleep undisturded by insect vampyres, because your wife is housekeeper.

Everybody who possibly can, ought to leave New York for the country sometime during the summer ; but to do so with advantage, the great aim should be recuperation, rest for the brain, rest for thc mind and physical repose. To accomplish these, get all the sleep you can during the night, be out in the open air every hour possible during daylight, in occupations which will compel the mind away from its usual routine of engagement in the pursuit of something which interests, instructs or profits. Exercise enough after breakfast in the open air to make you feel so hungry that bread and butter will taste sweet to you ; exercise enough in the open air after a noon-day dinner to digest that dinner most thoroughly ; eat not an atom between meals, and with a piece of cold bread and butter and a cup of any warm drink at sundown, pass the evening in joyous or instructive social intercourse. Spending a summer thus in the country, you will return to the city with a mental activity and a surplusage of general bodily health, which will fit you for another year's work in the great struggle for fame, fortune, or better still, usefulness to your kind.

